# Exploring Candidate Mechanisms of Change of EMIcompass, a Novel, Transdiagnostic Ecological Momentary Intervention for Prevention and Early Intervention in Youth: Findings From a Secondary Analysis of an Exploratory Randomized Controlled Trial

**DOI:** 10.1111/eip.70276

**Published:** 2026-11-13

**Authors:** Jessica Gugel, Isabell Paetzold Jaehme, Anita Schick, Dusan Hirjak, Jan R. Boehnke, Benjamin Boecking, Ulrich Reininghaus, Christian Rauschenberg

**Affiliations:** ^1^ Department of Public Mental Health Central Institute of Mental Health, Medical Faculty Mannheim, Heidelberg University Mannheim Germany; ^2^ Department of Psychiatry and Psychotherapy Central Institute of Mental Health, Medical Faculty Mannheim, Heidelberg University Mannheim Germany; ^3^ Faculty of Health University of Dundee Dundee UK; ^4^ Tinnitus Center Charité Universitätsmedizin Berlin Berlin Germany; ^5^ German Center for Mental Health (DZPG), Partner Site Mannheim‐Heidelberg‐Ulm Mannheim Germany; ^6^ ESRC Centre for Society and Mental Health and Social Epidemiology Research Group King's College London London UK; ^7^ Health Service and Population Research Department, Centre for Epidemiology and Public Health, Institute of Psychiatry, Psychology & Neuroscience King's College London London UK

**Keywords:** blended care, clinical staging, digital intervention, mobile health (mHealth) intervention, public mental health, transdiagnostic intervention

## Abstract

**Background:**

Ecological momentary interventions targeting transdiagnostic mechanisms are promising for prevention and early intervention, but evidence on their mechanisms of change is very limited.

**Objective:**

To explore indirect effects of EMIcompass, a transdiagnostic, compassion‐focused ecological momentary intervention, on mental health outcomes (i.e., psychological distress, clinical symptoms, quality of life) via candidate mechanisms of change (i.e., stress reactivity, emotional reactivity, threat anticipation, resilience and aberrant salience) in youth with early mental health problems.

**Methods:**

We conducted a secondary analysis of an exploratory, assessor‐blind randomized controlled trial, in which youth aged 14–25 years with early mental health problems were randomly allocated to experimental (EMIcompass + treatment as usual [TAU]) or control condition (TAU). Data was collected before randomization, at post‐intervention and at four‐week follow‐up.

**Results:**

*N* = 92 participants were recruited, of which *n* = 46 were allocated to the experimental condition. No signals of indirect effects of condition on any outcome through the five mechanisms (i.e., 95% confidence intervals included zero) were found. The magnitude of association, however, indicated that the association between EMIcompass and psychological distress may be mediated via pathways through threat anticipation (*β* = −0.12, 95% CI = −0.30–0.03). Further, we observed associations between changes in candidate mechanisms and mental health outcomes.

**Conclusions:**

Although no direct signals of mediation via candidate mechanisms were observed due to lack of power, condition‐linked shifts in our candidate mechanisms were associated with mental‐health outcomes. Larger mechanism‐powered trials are now required to delineate causal pathways and refine scalable prevention and early intervention strategies.

## 1 Introduction

Trajectories for early clinical phenotypes are often conceptualized through staging models that consider the frequency, intensity and severity of symptoms over time (Cornblatt et al. 2003; Mulraney et al. 2021; Yung and McGorry 1996; Yung et al. 2004). Recent clinical staging models posit that early signs and symptoms tend to be non‐specific, reflecting what has been termed an ‘extended transdiagnostic phenotype’ (van Os and Reininghaus 2016), a broad ‘clinical high at‐risk mental state’ (CHARMS) (Hartmann et al. 2019) or a ‘pluripotent risk state’ (Johannessen and McGorry 2010; McGorry 2013; Schulte‐Strathaus et al. 2025). These early presentations are thought to be associated with a range of potential exit syndromes later in life (Hartmann et al. 2019; McGorry 2013). Given the substantial disease burden that mental health conditions impose during adolescence (Erskine et al. 2015) and in later adulthood (Vigo et al. 2016), the need for effective prevention and early intervention strategies targeting youth becomes evident (Hartmann et al. 2021, 2019; McGorry 2013).

Despite the objective need for prevention and intervention for young individuals, access to professional help remains limited (Alonso et al. 2004). Digital mental health interventions may be a promising approach to help bridge this gap in demand and use (Borghouts et al. 2021) by providing low‐threshold support (Lehtimaki et al. 2021). Ecological momentary assessment (EMA; Stone and Shiffman 1994) can be deployed via apps as a structured digital diary technique to assess symptoms, mood and context in specific moments in time (Myin‐Germeys et al. 2018, 2009; Runyan and Steinke 2015; Shiffman et al. 2008; Stone and Shiffman 1994). Ecological momentary interventions (EMIs) leverage EMA data to deliver real‐time intervention when needed by using adaptive delivery schemes (Heron and Smyth 2010; Rauschenberg, Boecking, et al. 2021; Reininghaus, Depp, and Myin‐Germeys 2016). Building on this, EMIs show great potential to specifically target candidate mechanisms in the development of mental health conditions in daily life (Reininghaus et al. 2024; Schulte‐Strathaus et al. 2023).

As one important candidate mechanism, stress reactivity has been investigated in recent years and is defined as a more pronounced negative affective response to minor stressors in daily life (Myin‐Germeys and van Os 2007; Reininghaus, Kempton, et al. 2016; Vaessen et al. 2017; Wichers 2014). Stress reactivity has been found to be amplified by exposure to adverse childhood experiences, including childhood trauma, bullying victimization and negative life events (Collip et al. 2008; Paetzold, Gugel, et al. 2023; Paetzold et al. 2021; Rauschenberg et al. 2022, 2017; Rauschenberg, van Os, et al. 2021; Reininghaus, Gayer‐Anderson, et al. 2016) and may represent a risk and resilience mechanism in the development of various mental health outcomes (Rauschenberg et al. 2017; Reininghaus, Kempton, et al. 2016). In addition, other candidate mechanisms have been described (Ungar and Theron 2020), including aberrant salience (Reininghaus, Kempton, et al. 2016), emotional reactivity (Carver and Johnson 2018) and threat anticipation (Bentall et al. 2008; Paetzold, Gugel, et al. 2023). Previous research suggests that aberrant salience may represent an important candidate mechanism specifically for psychotic symptoms (Chun et al. 2020; Kapur 2003; Kvarta et al. 2021; Reininghaus, Kempton, et al. 2016; Reininghaus et al. 2019). In addition, empirical evidence has demonstrated the pivotal role of resilience as a protective factor in the development and persistence of mental health conditions (McLaughlin et al. 2020; Rutter 2007). Given the importance of risk and resilience in illness trajectories, these mechanisms may represent key targets for early intervention and prevention (Rauschenberg, Boecking, et al. 2021; Reininghaus, Depp, and Myin‐Germeys 2016; Reininghaus et al. 2023).

The first EMI directly targeting momentary stress reactivity in daily life and other candidate mechanisms is the ‘EMIcompass’ intervention, a transdiagnostic, compassion‐focused, hybrid ecological momentary intervention for improving resilience in youth with early mental health problems (Rauschenberg, Boecking, et al. 2021; Reininghaus et al. 2023; Schick et al. 2021). Findings from an exploratory randomized controlled trial (RCT) recently provided first evidence of feasibility of trial methodology and intervention delivery as well as initial signals of efficacy on candidate mechanisms (i.e., emotional reactivity, stress reactivity, threat anticipation, resilience and aberrant salience) and outcomes (i.e., quality of life and BPRS total scores) (Reininghaus et al. 2023). However, an exploration of putative indirect effects through which EMIcompass exerts its effects via candidate mechanisms on outcomes is pending. Using qualitative data collected alongside this exploratory RCT, a realist evaluation of the EMIcompass intervention suggested several putative pathways through which the intervention may impact the participants' soothing system, such as stress reactivity (Paetzold et al. 2025). Complementary to this qualitative work, the present secondary analysis of the exploratory RCT set out to explore candidate mechanisms through which EMIcompass may confer the previously reported outcomes.

The aim of this study was to investigate the indirect effects of the EMIcompass intervention on psychological distress, clinical symptoms and quality of life in youth with early mental health problems. We hypothesized that these outcomes are mediated by several candidate mechanisms of change, that is, stress reactivity, emotional reactivity, threat anticipation, resilience and aberrant salience. Specifically, we tested the following hypotheses:

*Effects of the EMIcompass intervention on psychological distress, clinical symptoms and quality of life as outcome variables at* 4*‐week follow‐up are mediated* via *change in stress reactivity (δpost‐intervention–baseline), while controlling for baseline levels of outcome variables, stress reactivity and socio‐demographic characteristics*.

*Effects of the EMIcompass intervention on psychological distress, clinical symptoms and quality of life as outcome variables at* 4*‐week follow‐up are mediated* via *change in emotional reactivity (δpost‐intervention–baseline), while controlling for baseline levels of outcome variables, emotional reactivity and socio‐demographic characteristics*.

*Effects of the EMIcompass intervention on psychological distress, clinical symptoms and quality of life as outcome variables at* 4*‐week follow‐up are mediated* via *change in threat anticipation (δpost‐intervention–baseline), while controlling for baseline levels of outcome variables, threat anticipation and socio‐demographic characteristics*.

*Effects of the EMIcompass intervention on psychological distress, clinical symptoms and quality of life as outcome variables at* 4*‐week follow‐up are mediated* via *change in resilience (δpost‐intervention–baseline) while controlling for baseline levels of outcome variables, resilience and socio‐demographic characteristics*.

*Effects of the EMIcompass intervention on psychological distress, clinical symptoms and quality of life as outcome variables at* 4*‐week follow‐up are mediated* via *change in aberrant salience (δpost‐intervention–baseline), while controlling for baseline levels of outcome variables, aberrant salience and socio‐demographic characteristics*.

*Effects of the EMIcompass intervention on prodromal symptoms as outcome at* 4*‐week follow‐up are mediated* via *change in aberrant salience (δpost‐intervention–baseline), while controlling for baseline levels of outcome variables, aberrant salience and socio‐demographic characteristics*.

## 2 Method

### 2.1 Study Design

In a secondary analysis of data collected in an exploratory, parallel‐group, assessor‐blind randomized control trial (RCT), youth aged 14–25 were randomized (50:50 ratio) to a control condition of treatment as usual (TAU) or an experimental condition (EMIcompass + TAU). The trial was conducted between August 2019 and September 2021. Data on candidate mechanisms and outcomes were collected before randomization (i.e., at ‘baseline’, *t*_0_), at the end of the 6‐week intervention period (i.e., at ‘post‐intervention’, *t*_1_) and at 4‐week follow‐up (i.e., ‘follow‐up’, *t*_2_). In addition, at all three time points (i.e., baseline, post‐intervention and follow‐up), EMA was used with eight assessments per day, scheduled at random within set time blocks, for six consecutive days to collect data on momentary candidate mechanisms and outcomes. The EMA questionnaire consisted in total of 47 items. The study was approved by the local Ethics Committee (2017‐602N‐MA). Further details on the study protocol (Schick et al. 2021), full intervention manual (Paetzold et al. 2022), as well as main (Reininghaus et al. 2023) and other findings (Paetzold et al. 2025; Paetzold, Schick, et al. 2023; Gugel et al. 2026) are presented elsewhere.

### 2.2 Participants

Youth aged 14–25 years with early mental health problems were recruited from mental health services at the Central Institute of Mental Health (CIMH), online advertisements, social media websites and local registries in Mannheim, Germany. Eligibility to participate was assessed using self‐report and observer‐rated measures (see Supplement 2 for further information). All participants (and for minors their caregivers or legal guardians) provided informed consent and received compensation for their time (range 85–175€) and travel expenses.

### 2.3 Measures

As sociodemographic variables, age, gender (female, male, non‐binary) and ethnicity (based on citizenship, country of birth, first language and family history) were assessed via self‐report at baseline. An overview of all outcome measures (i.e., psychological distress, clinical symptoms, quality of life and prodromal psychotic symptoms) and candidate mechanisms (i.e., stress reactivity, emotional reactivity, threat anticipation, resilience and aberrant salience) can be found in Table 1.

**TABLE 1:** Data collection and measures.

Outcome measures	
Psychological distress	We calculated a sum score for psychological distress that was assessed using the Kessler Psychological Distress Scale (K10; Kessler et al. 2002), a 10‐item questionnaire assessing psychological distress in the last month on a scale from 1 (= never) to 5 (= always).
Clinical symptoms	We calculated a total score across the 18 items of the Brief Psychiatric Rating Scale (BPRS; Kwan and Rickwood 2015), rated on a scale from 0 (not at all) to 4 (very strong).
Quality of life	We calculated a mean score across the 26 items of the brief version of the World Health Organization Quality of Life Instrument (WHOQOL‐BREF; Skevington et al. 2004; The WHOQOL‐Group 1998).
Prodromal psychotic symptoms	For our exploratory analyses, we used prodromal psychotic symptoms assessed with the 16 item Prodromal Questionnaire (PQ‐16) (Ising et al. 2012).
Putative mechanisms of change	
Stress reactivity	Stress reactivity (i.e., the association between stress and negative affect) was computed in a linear mixed model with momentary stress (mean score of event‐related, activity‐related, and social stress) as independent variable and negative affect as outcome variable. From this model, the generated fitted value was used for quantifying momentary stress reactivity (Paetzold et al. 2021; Reininghaus, Kempton, et al. 2016).
Emotional reactivity	Emotional reactivity (also referred to as negative affect) was assessed based on 6 established EMA items measuring negative affect with a 7‐point Likert‐scale ranging from 1 (= ‘not at all’) to 7 (= ‘very much’) (Reininghaus, Kempton, et al. 2016): ‘I feel anxious’, ‘I feel lonely’, ‘I feel insecure’, ‘I feel angry’, ‘I feel annoyed’, ‘I feel down’.
Threat anticipation	Threat anticipation was assessed with an EMA item as well as an established self‐report measure. In the EMA, participants were asked to think of what might happen in the next few hours and to rate the item ‘I think that something unpleasant will happen’ on a 7‐point Likert scale from 1 (= ‘not at all’) to 7 (= ‘very much’) (Reininghaus, Kempton, et al. 2016). It was also measured with the Threat Anticipation Measure (TAM, Corcoran et al. 2006), in which participants were asked to estimate the future likelihood of a list of negative/threatening and positive events (10 items in total) happening to themselves and to others.
Resilience	Resilience was assessed using one EMA item ‘I had difficulties to recover’ that participants were asked to rate on a 7‐point Likert scale from 1 (= ‘not at all’) to 7 (= ‘very much’) if they indicated that there was a negative event. The coding on this item was reversed, so that a higher rating on this item indicated a higher resilience.
Aberrant salience	In line with Reininghaus, Kempton, et al. (2016), aberrant salience was measured with three EMA items that were rated on a 7‐point Likert scale ranging from 1 (= ‘not at all’) to 7 (= ‘very much’): ‘Everything grabs my attention right now’, ‘Everything seems to have meaning right now’, ‘I notice things that I haven’t noticed before’.

*Note:* Further information on measures and data collection can be found in Reininghaus et al. (2023) and Schick et al. (2021).

### 2.4 Statistical Analyses

The current analysis was registered on the open science framework prior to accessing and analysing the data (https://osf.io/v67wt, (Gugel et al. 2024)). Deviations from the preregistration are reported in Supplement 1. Due to the exploratory nature of our secondary analyses, parameter estimates (95% confidence intervals) but no *p*‐values were obtained. To enhance comparability between measures of candidate mechanisms and outcomes, we used *z*‐standardized scores for all continuous variables.

Stata 17 (StataCorp 2021) was used for all exploratory analyses. Due to our small sample size we decided against a multilevel mediation structural equation modelling approach, according to previous recommendations (McNeish 2017). For all variables measured with EMA, we aggregated participants' EMA scores in daily life to a mean score for each assessment week (i.e., baseline, post‐intervention). We next calculated difference scores for all variables of candidate mechanisms (δpost‐intervention–baseline) to index change in the respective mechanism (i.e., stress reactivity, emotional reactivity, threat anticipation, resilience and aberrant salience). For each exploratory hypothesis, we fitted separate mediation models to examine whether effects of EMIcompass on the outcome variables (i.e., psychological distress, clinical symptoms, quality of life and for H6 prodromal symptoms) were mediated by change in the respective candidate mechanisms (i.e., stress reactivity, emotional reactivity, threat anticipation, resilience and aberrant salience). The outcome variables at 4‐week follow‐up were included in the models as dependent variables, change in the respective candidate mechanism (δpost‐intervention–baseline) as mediator and condition as independent variable. All analyses were adjusted for outcome variables, mediator variables, age and gender (3‐level factor) at baseline as potential confounders. We obtained parameter estimates for the main effects of condition and change in the respective candidate mechanism, on the one hand, on outcomes assessed at 4‐week‐follow‐up, on the other, as well as for main effects of condition on change in the respective candidate mechanisms. The total effects of condition on our outcomes were apportioned into direct and indirect effects through change in candidate mechanisms. All indirect effects were computed via the product of coefficients strategy. We used bootstrapping for all mediation analyses according to recommendations by Preacher et al. (2007). As a measure of effect size, we computed the proportion mediated, i.e., the ratio of the indirect effect to the total effect (MacKinnon et al. 2007; Shrout and Bolger 2002). For further exploratory analyses, we repeated the abovementioned analyses using (a) outcome data measured at post‐intervention (see Supplement 3) and (b) using the different subscales of the WHOQOL‐BREF as outcome measures (i.e., physical health, psychological health, social relationships and environment) (see Supplement 4). To reflect consistency of our analyses with results of the EMIcompass RCT (Reininghaus et al. 2023), we performed sensitivity analyses using different conceptualisations of change in stress reactivity and regression analyses to examine the effect of condition on stress reactivity (see Supplement 5). Finally, a correlation matrix of all candidate mechanisms of change and outcome variables in our main analyses was calculated and multicollinearity was evaluated by calculating variance inflation factors (see Supplement 6).

All analyses were conducted following the intention‐to‐treat (ITT) principle (Gupta 2011; Montori and Guyatt 2001), incorporating data from all participants into the analysis including those who have low adherence to, or who dropped out from the intervention. Maximum likelihood estimation was applied, allowing for the use of all available data under the relatively unrestrictive assumption that data is missing at random and all variables associated with missing values are included in the model.

## 3 Results

### 3.1 Basic Sample Characteristics

Table 2 displays an overview of basic sample characteristics of the ITT sample. The full sample consisted of *N* = 92 participants (i.e., *N* = 46 experimental condition, *N* = 46 control condition). The mean age was 21.65 years (SD = 2.84, range 14–25). Of all participants, approximately 22% were identified as having an ethnic minority background. The majority of our sample was classified as Stage 1a (psychological distress, 57%), 30% were classified as Stage 1b (CHARMS) and 13% were classified as Stage 2 (first episode of a severe mental health condition).

**TABLE 2:** Basic sample characteristics of the full sample.

	Full sample (*N* = 92)	*n*	Experimental condition (*N* = 46)	*n*	Experimental condition (*N* = 46)	*n*
Age (years), mean (SD)	21.67 (2.49)		21.30 (2.84)		22.04 (2.05)	
Gender, *n* (%)						
Female	67 (73%)		35 (76%)		32 (70%)	
Male	25 (27%)		11 (24%)		14 (30%)	
Other	0 (0%)		0 (0%)		0 (0%)	
Ethnicity, *n* (%)						
White majority	72 (78.3%)		32 (69.6%)		40 (87.0%)	
Minority						
Mixed white majority/white other	5 (5.4%)		3 (6.5%)		2 (4.4%)	
White other	5 (5.4%)		4 (8.7%)		1 (2.2%)	
Turkish	4 (4.4%)		3 (6.5%)		1 (2.2%)	
Mixed other	3 (3.3%)		2 (4.4%)		1 (2.2%)	
Middle east	2 (2.2%)		1 (2.2%)		1 (2.2%)	
Asian	1 (1.1%)		1 (2.2%)		0 (0.0%)	
Clinical staging, *n* (%)						
Stage 1a	52 (56.5%)		26 (56.5%)		26 (56.5%)	
Stage 1b	28 (30.4%)		13 (28.3%)		15 (32.6%)	
Stage 2	12 (13.0%)		7 (15.2%)		5 (10.9%)	
Psychological distress, mean (SD)						
At baseline	28.08 (5.12)	92	28.20 (5.08)	46	27.96 (5.21)	46
At post‐intervention	23.76 (6.53)	89	24.11 (6.55)	45	23.41 (6.56)	44
At follow‐up	22.50 (6.71)	88	22.73 (7.16)	45	22.26 (6.27)	43
Clinical symptoms, mean (SD)						
At baseline	31.40 (5.14)	92	32.00 (5.07)	46	30.80 (5.19)	46
At post‐intervention	31.08 (5.58)	89	30.87 (5.60)	45	31.30 (5.62)	44
At follow‐up	31.11 (4.97)	88	31.82 (5.49)	45	30.37 (4.30)	43
Overall quality of life, mean (SD)						
At baseline	14.12 (1.63)	53	13.92 (1.49)	25	14.30 (1.74)	28
At post‐intervention	14.75 (1.80)	67	14.95 (1.86)	33	14.57 (1.76)	34
At follow‐up	15.07 (1.75)	73	15.18 (1.63)	37	14.96 (1.88)	36
Prodromal symptoms, mean (SD)						
At baseline	2.69 (2.52)	91	3.02 (2.76)	46	2.36 (2.23)	45
At post‐intervention	3.05 (3.04)	88	3.50 (3.32)	44	2.59 (2.69)	44
At follow‐up	2.57 (2.55)	87	3.11 (3.00)	44	2.02 (1.87)	43
Emotional reactivity, mean (SD)						
At baseline	2.16 (0.69)	92	2.21 (0.72)	46	2.10 (0.66)	46
At post‐intervention	2.05 (0.83)	87	2.07 (0.89)	44	2.04 (0.78)	43
Stress reactivity, mean (SD)						
At baseline	2.09 (0.68)	92	2.09 (0.68)	46	2.09 (0.68)	46
At post‐intervention	2.05 (0.68)	87	2.08 (0.72)	44	2.02 (0.64)	43
Threat anticipation, mean (SD)						
At baseline	2.45 (1.00)	92	2.42 (0.96)	46	2.48 (1.06)	46
At post‐intervention	2.30 (1.11)	87	2.17 (1.04)	44	2.43 (1.19)	43
Threat anticipation (non‐EMA), mean (SD)						
At baseline	11.82 (4.28)	92	12.48 (4.44)	46	11.15 (4.05)	46
At post‐intervention	12.21 (5.19)	89	12.18 (5.23)	45	12.25 (5.21)	44
Resilience, mean (SD)						
At baseline	3.71 (1.26)	83	3.60 (1.38)	43	3.82 (1.11)	40
At post‐intervention	4.15 (1.44)	69	4.37 (1.53)	35	3.92 (1.33)	34
Aberrant salience, mean (SD)						
At baseline	1.51 (0.61)	92	1.56 (0.61)	46	1.46 (0.63)	46
At post‐intervention	1.46 (0.80)	87	1.24 (0.42)	44	1.68 (1.01)	43
Completed EMA prompts (compliance %)						
At baseline	39.21 (6.18, 82%)	92	39.75 (5.49, 83%)	46	38.69 (6.83, 81%)	46
At post‐intervention	33.71 (10.26, 70%)	89	34.76 (8.87, 72%)	45	32.65 (11.51, 68%)	44

*Note:* From the full sample of the EMIcompass trial (*N* = 92), 46 participants were in the experimental condition. Sample sizes varied over the different scales due to missing values. See Reininghaus et al. (2023) for the main results of the EMIcompass trial and Paetzold et al. (2022) for further information on baseline characteristics.

Abbreviation: SD, standard deviation.

### 3.2 Candidate Mechanism (1): Stress Reactivity

Table 3 shows findings on stress reactivity as candidate mechanism. We observed no signals of indirect effects via pathways through change in stress reactivity (*t*_1_–*t*_0_). There was, however, an association of change in stress reactivity with psychological distress (*β* = 0.72, 95% CI = 0.07–1.50), such that a greater increase in stress reactivity from *t*_0_ to *t*_1_ was associated with higher psychological distress.

**TABLE 3:** The indirect effects of experimental condition on psychological distress, clinical symptoms and quality of life at four‐week follow‐up via pathways through change in stress reactivity^a^ ^,^ ^b^.

	Psychological distress			Clinical Symptoms			Quality of Life		
	aβ^c^	CI	P_M_ ^d^	aβ	CI	P_M_	aβ	CI	P_M_
Total effect	−0.05	−0.45–0.33		0.24	−0.16–0.64		0.32	−0.30–0.86	
Direct effect of condition on outcome	−0.05	−0.41–0.32		0.24	−0.16–0.66		0.33	−0.29–0.88	
Effect of condition on mediator	−0.00	−0.12–0.12		0.00	−0.12–0.13		0.03	−0.08–0.15	
Effect of mediator on outcome	0.72	0.07–1.50		−0.15	−0.96–0.78		−0.23	−1.56–0.89	
Indirect effect	−0.00	−0.11–0.09	0.03	−0.00	−0.08–0.05	0.01	−0.01	−0.16–0.04	0.02

^a^ *n* = 87 for psychological distress and clinical symptoms and 52 for quality of life.

^b^ All analyses were adjusted for age, gender and baseline levels of outcomes and mediator.

^c^ Standardized regression coefficient.

^d^ Proportion mediated.

### 3.3 Candidate Mechanism (2): Emotional Reactivity

In Table 4, findings on emotional reactivity as a candidate mechanism are shown. No signals of indirect effects via pathways through change in emotional reactivity (*t*_1_–*t*_0_) were observed for all three candidate outcomes. There was, however, an association of change in emotional reactivity with clinical symptoms (β = 0.38, 95% CI = 0.15–0.78).

**TABLE 4:** The indirect effects of experimental condition on psychological distress, clinical symptoms and quality of life at four‐week follow‐up via pathways through change in emotional reactivity^a^ ^,^ ^b^.

	Psychological distress			Clinical Symptoms			Quality of Life		
	aβ^b^, ^c^	CI	P_M_ ^d^	aβ	CI	P_M_	aβ	CI	P_M_
Total effect	−0.06	−0.46–0.38		0.19	−0.22–0.64		0.37	−0.19–0.91	
Direct effect of condition on outcome	0.01	−0.35–0.40		0.24	−0.15–0.67		0.32	−0.26–0.89	
Effect of condition on mediator	−0.12	−0.37–0.19		−0.13	−0.39–0.16		−0.18	−0.49–0.13	
Effect of mediator on outcome	0.61	−0.35–0.95		0.38	0.15–0.78		−0.27	−0.89–0.14	
Indirect effect	−0.07	−0.24–0.10	1.21^e^	−0.05	−0.24–0.04	0.25	0.05	−0.02–0.32	0.13

^a^ *n* = 87 for psychological distress and clinical symptoms and 52 for quality of life.

^b^ All analyses were adjusted for age, gender and baseline levels of outcomes and mediator.

^c^ Standardized regression coefficient.

^d^ Proportion mediated.

^e^ Exceeds 1.0 in case of suppression effects, that is, if direct effect is opposite in sign to the indirect effect (Shrout and Bolger 2002).

### 3.4 Candidate Mechanism (3): Threat Anticipation

In Table 5, findings on threat anticipation as candidate mechanism are shown. Although the CI for the indirect effect of change in threat anticipation measured with aggregated EMA on psychological distress includes zero, the magnitude of association indicates initial signals for a potential indirect effect (*β* = −0.12, 95% CI = −0.30–0.03). There were no signals of indirect effects via pathways through change in threat anticipation measured with non‐EMA for the three outcomes. We further found initial signals for an effect of condition on change in threat anticipation measured with aggregated EMA (in the model with quality of life as outcome: *β* = −0.32, 95% CI = −0.69–‐0.03). Compared with the control condition, there was a stronger decrease in momentary threat anticipation from *t*_0_ to *t*_1_ in the experimental condition. Also, we observed associations for change in threat anticipation with psychological distress (*β* = 0.59, 95% CI = 0.26–0.88) and clinical symptoms (*β* = 0.48, 95% CI = 0.12–0.88).

**TABLE 5:** The indirect effects of experimental condition on psychological distress, clinical symptoms and quality of life at four‐week follow‐up via pathways through change in threat anticipation^a^ ^,^ ^b^.

	Psychological distress			Clinical symptoms			Quality of life		
	aβ^c^	CI	P_M_ ^d^	aβ	CI	P_M_	aβ	CI	P_M_
Threat anticipation measured with EMA									
Total effect	−0.02	−0.44–0.40		0.25	−0.16–0.70		0.38	−0.27–0.91	
Direct effect of condition on outcome	0.09	−0.30–0.51		0.36	−0.07–0.85		0.29	−0.41–0.89	
Effect of condition on mediator	−0.20	−0.43–0.05		−0.22	−0.49–0.03		−0.32	−0.69–−0.03	
Effect of mediator on outcome	0.59	0.26–0.88		0.48	0.12–0.88		−0.27	−0.79–0.21	
Indirect effect	−0.12	−0.30–0.03	4.78^e^	−0.10	−0.33–0.00	0.41	0.09	−0.02–0.41	0.23
Threat anticipation measured with TAM									
Total effect	−0.05	−0.39–0.38		0.21	−0.16–0.64		0.41	−0.20–0.94	
Direct effect of condition on outcome	−0.05	−0.39–0.39		0.23	−0.16–0.65		0.41	−0.21–0.92	
Effect of condition on mediator	−0.13	−0.53–0.24		−0.15	−0.56–0.23		0.01	−0.46–0.50	
Effect of mediator on outcome	0.03	−0.16–0.19		0.08	−0.16–0.28		−0.24	−0.59–0.08	
Indirect effect	−0.00	−0.07–0.02	0.07	−0.01	−0.16–0.02	0.06	−0.00	−0.20–0.11	0.01

^a^ *n* = 87 for the analyses with momentary threat anticipation and psychological distress and clinical symptoms and 52 for quality of life, *n* = 89 for the analyses with threat anticipation measured with TAM and psychological distress and clinical symptoms and 52 for quality of life.

^b^ All analyses were adjusted for age, gender and baseline levels of outcomes and mediator.

^c^ Standardized regression coefficient.

^d^ Proportion mediated.

^e^ Exceeds 1.0 in case of suppression effects, that is, if direct effect is opposite in sign to the indirect effect (Shrout and Bolger 2002).

### 3.5 Candidate Mechanism (4): Resilience

In Table 6, findings on resilience as a candidate mechanism are shown. No initial signals of indirect effects via pathways through change in resilience (*t*_1_–*t*_0_) for the three outcomes were observed. There was, however, an association for change in resilience with quality of life (*β* = 0.35, 95% CI = 0.02–0.70), such that a stronger increase in resilience was associated with higher quality of life.

**TABLE 6:** The indirect effects of experimental condition on psychological distress, clinical symptoms and quality of life at four‐week follow‐up via pathways through change in resilience^a^ ^,^ ^b^.

	Psychological distress			Clinical Symptoms			Quality of Life		
	aβ^c^	CI	P_M_ ^d^	aβ	CI	P_M_	aβ	CI	P_M_
Total effect	−0.24	−0.68–0.21		0.26	−0.18–0.89		0.44	−0.07–0.89	
Direct effect of condition on outcome	−0.23	−0.72–0.24		0.28	−0.24–0.95		0.35	−0.13–0.83	
Effect of condition on mediator	−0.21	−0.20–0.65		0.24	−0.22–0.62		0.24	−0.17–0.70	
Effect of mediator on outcome	−0.09	−0.31–0.19		−0.11	−0.53–0.35		0.35	0.02–0.70	
Indirect effect	−0.02	−0.16–0.05	0.07	−0.03	−0.29–0.07	0.11	0.08	−0.02–0.37	0.19

^a^ *n* = 65 for psychological distress and clinical symptoms and 39 for quality of life.

^b^ All analyses were adjusted for age, gender and baseline levels of outcomes and mediator.

^c^ Standardized regression coefficient.

^d^ Proportion mediated.

### 3.6 Candidate Mechanism (5): Aberrant Salience

In Table 7, findings on aberrant salience as candidate mechanism are shown. There were no initial signals for indirect effects via pathways through change in aberrant salience (*t*_1_–*t*_0_) for the three outcomes. However, we did observe an effect of condition on change in aberrant salience in all models with psychological distress (*β* = −0.54, 95% CI = −0.90–−0.29), clinical symptoms (*β* = −0.56, 95% CI = −0.88–−0.28) and quality of life (*β* = −0.52, 95% CI = −1.05–−0.13) as outcomes. Also, we found an association for change in aberrant salience with clinical symptoms (*β* = 0.23, 95% CI = 0.01–0.47).

**TABLE 7:** The indirect effects of experimental condition on psychological distress, clinical symptoms and quality of life at four‐week follow‐up via pathways through change in aberrant salience^a^ ^,^ ^b^.

	Psychological distress			Clinical Symptoms			Quality of Life		
	aβ^c^	CI	P_M_ ^d^	aβ	CI	P_M_	aβ	CI	P_M_
Total effect	−0.03	−0.41–0.37		0.21	−0.20–0.66		0.41	−0.22–0.89	
Direct effect of condition on outcome	0.09	−0.29–0.50		0.34	−0.10–0.89		0.35	−0.34–0.90	
Effect of condition on mediator	−0.54	−0.90–−0.29		−0.56	−0.88–−0.28		−0.52	−1.05–−0.13	
Effect of mediator on outcome	0.22	−0.06–0.56		0.23	0.01–0.47		−0.11	−0.49–0.17	
Indirect effect	−0.12	−0.31–0.03	4.27^e^	−0.13	−0.28–0.02	0.64	0.06	−0.07–0.28	0.15

^a^ *n* = 87 for psychological distress and clinical symptoms and 52 for quality of life.

^b^ All analyses were adjusted for age, gender and baseline levels of outcomes and mediator.

^c^ Standardized regression coefficient.

^d^ Proportion mediated.

^e^ Exceeds 1.0 in case of suppression effects, that is, if direct effect is opposite in sign to the indirect effect (Shrout and Bolger 2002).

### 3.7 Indirect Effects on Prodromal Symptoms via Change in Aberrant Salience

In Table 8, findings on aberrant salience as a candidate mechanism for prodromal symptoms are shown. We found no signals of indirect effects via pathways through change in aberrant salience (*t*_1_–*t*_0_) for prodromal symptoms.

**TABLE 8:** The indirect effects of experimental condition on prodromal symptoms at four‐week follow‐up via pathways through change in aberrant salience^a^ ^,^ ^b^.

	Prodromal symptoms		
	aβ^c^	CI	P_M_ ^d^
Total effect	0.26	−0.02–0.71	
Direct effect of condition on outcome	0.23	−0.07–0.71	
Effect of condition on mediator	−0.55	−0.88–−0.30	
Effect of mediator on outcome	−0.06	−0.34–0.25	
Indirect effect	0.04	−0.13–0.19	0.14

^a^ *n* = 86.

^b^ All analyses were adjusted for age, gender and baseline levels of outcomes and mediator.

^c^ Standardized regression coefficient.

^d^ Proportion mediated.

## 4 Discussion

### 4.1 Principal Findings

This study advanced on previous research by investigating whether changes in candidate mechanisms mediated the effect of EMIcompass—a hybrid, compassion‐focused ecological momentary intervention—on candidate mental health outcomes in youth with early mental health problems. There were initial signals indicating a potential indirect effect of threat anticipation measured with aggregated EMA, but no initial signals of indirect effects of EMIcompass on candidate outcomes via stress reactivity, emotional reactivity, resilience and aberrant salience. Further, our findings signalled patterns of associations between EMIcompass, candidate mechanisms and outcomes. Specifically, we observed signals of beneficial effects of EMIcompass on reductions in threat anticipation measured with aggregated EMA and aberrant salience. We also observed signals of associations between candidate mechanisms and outcomes: that is, signals of reductions in stress reactivity and threat anticipation measured with aggregated EMA, on the one hand, to be associated with lower psychological distress, on the other; signals of reductions in emotional reactivity, threat anticipation, aberrant salience to be associated with less severe clinical symptoms; and signals of increases in resilience to be associated with higher quality of life. Although no mediation effects were found, the association patterns suggest our candidate mechanisms as potential transdiagnostic, momentary psychological mechanisms for mental health outcomes.

### 4.2 Methodological Considerations

Several methodological considerations should be taken into account before interpreting reported findings. First, the exploratory RCT was not powered for the current mediation analyses, so effects should be considered in terms of magnitude of associations rather than statistical significance. However, some of our findings suggest initial signals of effects that may inform planning of a confirmatory RCT. Second, in contrast to the main analyses from this exploratory RCT, stress reactivity and other momentary candidate mechanisms of change were operationalized by aggregating EMA data to composite mean scores for each 6‐day EMA period. This yielded a loss of intra‐individual variability and temporal dynamics in the data, which limits power, interpretation on real‐time fluctuations and short‐term changes or intervention effects (Oleson et al. 2022; Shiffman et al. 2008). As a consequence of the loss of intra‐individual variability, aggregated scores are subject to sampling error which may affect the precision of estimates (Stone et al. 2023). Moreover, aggregated data may not fully represent participants' experiences if EMA missingness follows systemic patterns. Although the high EMA compliance in the current analyses may limit this risk (see Table 2), potential bias cannot be entirely excluded. In adequately powered future studies, statistical approaches adopted for modelling signals of efficacy on momentary stress reactivity directly using mixed effects regression models to harness intensive longitudinal data on moment‐to‐moment variation in stress reactivity generated by multilevel structural equation modelling (MSEM) are preferable (Preacher et al. 2010). However, the sample size of the current exploratory RCT was not sufficient to apply these more complex modelling approaches (McNeish 2017). Third, it is important to critically evaluate the sample selection. The sample of the EMIcompass trial predominantly consisted of female individuals that may limit generalizability of findings to young male and non‐binary individuals. Differences in the prevalence of symptoms and diagnoses by gender may have partly contributed to this sample distribution, with depression and anxiety being more prevalent in females (Afifi 2007; Young et al. 2002). Fourth, intercorrelations between variables should be considered. Overall, we found small to moderate intercorrelations (between *r* = −0.57 and *r* = 0.63) between variables (see Supplement 6). Some candidate mechanisms of change shared significant overlap, which is conceptually plausible given prior work indicating empirical relations among related constructs (e.g., change in emotional reactivity with change in stress reactivity (Reininghaus, Kempton, et al. 2016)). These variables were, however, not entered simultaneously into the same regression models. Instead, candidate mechanisms were evaluated in separate models. Correlations between candidate mechanisms and outcomes were generally smaller than the correlations among mechanisms and no patterns indicated problematic collinearity for estimated models (see Supplement 6). Last, the EMIcompass RCT was conducted during the COVID‐19 pandemic, when restrictions on social contacts, heightened health‐related stress or a shift to working from home might have affected participants' mental health (Rauschenberg, Schick, et al. 2021; Ravens‐Sieberer et al. 2022).

### 4.3 Comparison to Previous Research

Previous meta‐analytic evidence indicates that compassion‐focused therapy (CFT) enhances compassion‐related outcomes and reduces a range of symptom domains (Kirby et al. 2017; Millard et al. 2023; Petrocchi et al. 2024), consistent with initial efficacy signals from the EMIcompass trial on mental health outcomes and candidate mechanisms (Reininghaus et al. 2023). Mediation analyses based on longitudinal data allow us to advance on this to explore temporality as an important criterion for causal inference and to establish mechanisms of change (Kazdin 2007, 2009; Susser 1991). The ecological interventionist causal model approach (Reininghaus, Depp, and Myin‐Germeys 2016) extends causal testing into participants' everyday lives, using EMIs such as EMIcompass to actively target candidate mechanisms. In the current analyses, we focused on changes in the candidate mechanisms using EMA data aggregated over the 6‐day EMA period, whereas the EMIcompass RCT identified signals of efficacy of EMIcompass on momentary candidate mechanisms operationalized at the level of individual EMA observations. This, together with limited power, explains discrepancies in interpreting our findings versus findings of the EMIcompass RCT.

Although the EMIcompass RCT (Reininghaus et al. 2023) found an effect of condition on momentary stress reactivity, in our analyses no effect of condition on change in stress reactivity emerged, likely due to reduced statistical power and focus on change rather than level effects. Our sensitivity analyses suggest that the effects of condition on change in stress reactivity vary depending on the time point analysed, consistent with the EMIcompass RCT, in which condition effects were strongest at follow‐up (see Supplement 5). This might indicate that our chosen time frame may not be adequate to fully capture the effects of EMIcompass. The association between reductions in stress reactivity and lower psychological distress aligns with results derived from qualitative interviews from participants as part of the EMIcompass process evaluation indicating that the intervention increased their competence in handling challenging situations, which they linked to improved well‐being and reductions in momentary symptom burden (Paetzold et al. 2025). These findings are further supported by studies demonstrating associations of individuals' stress reactivity with various mental health outcomes (Reininghaus, Kempton, et al. 2016; Vaessen et al. 2017). We did not find, however, associations between change in stress reactivity and clinical symptoms and quality of life. Given prior evidence suggesting that stress reactivity may be more strongly linked to specific symptom domains, such as psychotic symptoms (Devylder et al. 2013), future research should examine whether distinct pathways of change exist depending on individuals' symptom profiles. Importantly, our longitudinal results provide additional support to the primary findings of the EMIcompass RCT by identifying stress reactivity as a candidate mechanism associated with psychological distress over time (Reininghaus et al. 2023).

Although no signals for indirect effects via emotional reactivity were found, changes in emotional reactivity were associated with reduced clinical symptoms. This is consistent with findings from the EMIcompass process evaluation linking reduced negative affect to enhanced well‐being, hope and self‐esteem (Paetzold et al. 2025), as well as with prior research indicating short‐term effects of a brief CFT intervention on affect (Lincoln et al. 2013). CFT emphasizes coping with negative emotions rather than merely experiencing them (Gilbert 2009, 2013, 2014), consistent with previous findings that emotional tolerance may mediate the relationship between self‐compassion and depressive symptoms (Diedrich et al. 2017) and that improved emotion regulation predicted lower levels of clinical outcomes in the EMIcompass RCT (Paetzold, Schick, et al. 2023). Accordingly, by operationalizing emotional reactivity as the expression of negative affect, possible effects may have been obscured.

In line with previous research linking threat anticipation and psychopathology (Grupe and Nitschke 2013; Klippel et al. 2017; Paetzold, Gugel, et al. 2023; Reininghaus, Kempton, et al. 2016), a decrease in aggregated momentary threat anticipation was associated with lower levels of psychological distress and clinical symptoms in our analyses. This finding also aligns with experimental studies showing that compassion‐focused imagery can activate the parasympathetic nervous system, as evidenced by increases in heart rate variability and decreases in cortisol levels (Rockliff et al. 2008; Steffen et al. 2021), thereby reducing threat‐related arousal. However, threat anticipation as measured with non‐EMA did not show significant associations with outcomes, possibly due to greater sensitivity to change of momentary over retrospective assessments (Moore et al. 2016; Moskowitz and Young 2006; Myin‐Germeys et al. 2018).

Consistent with earlier studies linking resilience to quality of life (Chuang et al. 2023; Lawford and Eiser 2001; Reininghaus et al. 2023; Simón‐Saiz et al. 2018), our findings signalled an association between change in resilience and quality of life, further highlighting the role of resilience as a potential mechanism of change. Our additional exploratory analyses examining potential differences in candidate mechanisms across the subscales of quality of life further support this association between resilience and quality of life, with particularly strong effects observed for the psychological health subscale (see Supplement 4). This is notable given that enhancing resilience in youth was a central goal of EMIcompass and evident in previously reported findings on initial efficacy signals of EMIcompass on enhanced momentary resilience (Reininghaus et al. 2023). Pathways through which resilience may impact on mental health outcomes might also differ depending on symptom profiles, with prior findings suggesting that resilience may mediate the effect of self‐compassion on depressive symptoms but not on anxiety (Pérez‐Aranda et al. 2021).

Previous research has emphasized the relevance of aberrant salience, particularly in relation to paranoia (Kapur 2003), including findings based on EMA data (Reininghaus, Kempton, et al. 2016; Reininghaus et al. 2019; So et al. 2018). In our study, changes in aberrant salience were associated with clinical symptoms, although no association was found with prodromal symptoms—likely due to limited statistical power and restricted variance in this outcome. Existing experimental work has shown that CFT techniques may reduce paranoid ideation by influencing responses to negative affect (Lincoln et al. 2013).

Exploratory analyses with outcome data measured at post‐intervention and sensitivity analyses with different conceptualisation of change in stress reactivity (Supplements 3 and 5) showed similar patterns, with minor differences consistent with variations of effects of EMIcompass on candidate mechanisms and outcomes depending on the time point (Reininghaus et al. 2023). To better understand change, it is crucial to consider temporal dynamics and complex pathways in which interventions may unfold their effects (Berli et al. 2021), including examining effects on the micro‐level (Gugel et al. 2026) and longer‐term trajectories. While CFT has demonstrated efficacy across a broad range of mental health conditions, including hard‐to‐treat populations such as individuals with personality disorders (Craig et al. 2020; Leaviss and Uttley 2015), it is likely that mechanisms of change differ depending on aspects such as symptom profiles (Pérez‐Aranda et al. 2021), personality factors (Kelly et al. 2010, 2009; Shapira and Mongrain 2010), or duration of delivery (Craig et al. 2020).

## 5 Conclusion

This study investigated changes in stress reactivity, emotional reactivity, threat anticipation, resilience and aberrant salience as candidate mechanisms of change in the EMIcompass trial. We found initial signals of indirect effects via threat anticipation, but no evidence for indirect effects of condition on outcomes via stress reactivity, emotional reactivity, resilience and aberrant salience. However, several associations of candidate mechanisms with mental health outcomes at follow‐up emerged, further emphasizing their potential relevance in hybrid digital mental health interventions. Given limited power, firm conclusions on the role of EMIcompass to bring about change cannot be drawn from the current study. Micro‐randomized trials are required to allow for a more nuanced understanding of mechanisms of change and for whom and under which circumstances they operate. This may in turn help to inform the development of more personalized and adaptive interventions (Nye et al. 2023; Reininghaus 2018; Schulte‐Strathaus et al. 2023). Elucidating these dynamic pathways will ultimately enable the development of more precise, mechanism‐targeted digital mental health strategies for mental‐health promotion, prevention and early intervention—strategies that may be capable of shifting the population distribution of mental health towards greater resilience and well‐being (Reininghaus et al. 2022).

## Funding

This work was supported by the German Research Foundation (DFG) [grant number 389626655] and DFG Heisenberg professorship [grant number 389624707] to Ulrich Reininghaus. Endorsed by the German Center for Mental Health (DZPG).

## Conflicts of Interest

The authors declare no conflicts of interest.

## Supporting information


**Table S1:** The indirect effects of experimental condition on psychological distress, clinical symptoms and quality of life at post‐intervention via pathways through change in stress reactivity^a,b^.
**Table S2:** The indirect effects of experimental condition on psychological distress, clinical symptoms and quality of life at post‐intervention via pathways through change in emotional reactivity^a,b^.
**Table S3:** The indirect effects of experimental condition on psychological distress, clinical symptoms and quality of life at post‐intervention via pathways through change in threat anticipation^a,b^.
**Table S4:** The indirect effects of experimental condition on psychological distress, clinical symptoms and quality of life at post‐intervention via pathways through change in resilience^a,b^.
**Table S5:** The indirect effects of experimental condition on psychological distress, clinical symptoms and quality of life at post‐intervention via pathways through change in aberrant salience^a,b^.
**Table S6:** The indirect effects of experimental condition on the subscales of quality of life at four‐week follow‐up via pathways through change in stress reactivity^a,b^.
**Table S7:** The indirect effects of experimental condition on the subscales of quality of life at four‐week follow‐up via pathways through change in emotional reactivity^a,b^.
**Table S8:** The indirect effects of experimental condition on the subscales of quality of life at four‐week follow‐up via pathways through change in threat anticipation^,b^.
**Table S9:** The indirect effects of experimental condition on the subscales of quality of life at four‐week follow‐up via pathways through change in resilience^a,b^.
**Table S10:** The indirect effects of experimental condition on the subscales of quality of life at four‐week follow‐up via pathways through change in aberrant salience^,b^.
**Table S11:** The indirect effects of experimental condition on psychological distress, clinical symptoms and quality of life at four‐week follow‐up via pathways through change in stress reactivity (δ4‐week follow‐up–baseline)^a,b^.
**Table S12:** The indirect effects of experimental condition on psychological distress, clinical symptoms and quality of life at four‐week follow‐up via pathways through change in stress reactivity (δ4‐week follow‐up–post‐intervention)^a,b^.
**Table S13:** Effects of condition on stress reactivity measured at post‐intervention and follow‐up.
**Table S14:** Intercorrelation matrix for all candidate mechanisms of change^a^ and outcome variables^b^ in our main analyses.

## Data Availability

Data acquired during the EMIcompass trial will not be publicly available. The data, protocol and statistical code are available from the corresponding author upon reasonable request.
