# Supplementary material for: Exploring Candidate Mechanisms of Change of EMIcompass, a Novel, Transdiagnostic Ecological Momentary Intervention for Prevention and Early Intervention in Youth: Findings From a Secondary Analysis of an Exploratory Randomized Controlled Trial
Source: Early Interv Psychiatry. 2026 Nov 13;20(11):e70276. doi: 10.1111/eip.70276 (PMC13768146; doi:10.1111/eip.70276)
Supplement: Supplementary file 1 [file EIP-20-0-s001.docx]

**Supplement**

Gugel J, Rauschenberg C, Paetzold I, Schick A, Hirjak D, Boehnke J, Boecking B, Reininghaus U. Exploring Candidate Mechanisms of Change of EMIcompass, a Novel, Transdiagnostic Ecological Momentary Intervention for Prevention and Early Intervention in Youth: Findings from an Exploratory Randomized Controlled Trial.

Table of Contents

[**Supplement 1: Deviations from the preregistration**	2](#_Toc221624372)

[**Supplement 2: Eligibility criteria**	3](#_Toc221624373)

[**Supplement 3: Exploratory analyses of indirect effects of experimental condition on outcomes at post-intervention through candidate mechanisms**	4](#_Toc221624374)

[**Supplement 4: Exploratory analyses with the different subscales of the WHOQOL-BREF**	7](#_Toc221624375)

[**Supplement 5: Sensitivity analyses with different conceptualisations of change in stress reactivity**	12](#_Toc221624376)

[**Supplement 6: Intercorrelations and Multicollinearity Diagnostics**	15](#_Toc221624377)

[**References**	16](#_Toc221624378)

# **Supplement 1: Deviations from the preregistration**

Gugel, J., Rauschenberg, C., Paetzold, I., Schick, A., Hirjak, D., Banaschewski, T., Meyer-Lindenberg, A., Boehnke, J. R., Boecking, B., & Reininghaus, U. (2024). Examining Putative Mechanisms of Change in a Novel, Transdiagnostic Ecological Momentary Intervention for Prevention and Early Intervention in Youth: Findings from the EMIcompass trial. Open Science Framework - Preregistration. <https://doi.org/https://doi.org/10.17605/OSF.IO/V67WT>

**Deviations:**

- Gender was used preregistered as a 3-level factor but was used as a 2-level factor in the analyses as no participant in the sample identified as “other”.
- In our preregistration, we did not specify that we will use bootstrapping for all mediation analyses as well as that we will compute the proportion mediated to measure effect size. This was later decided based on recommendations in the literature as stated in the manuscript.
- We added the sensitivity analyses in Supplement 5 after our analyses to improve our understanding of the data and to place our findings in the broader context of EMIcompass.
- We conducted exploratory analyses using the different subscales of the WHOQOL-BREF (i.e., physical health, psychological health, social relationships, and environment) as outcomes to better illustrate of domain-specific changes (see Supplement 4).

# **Supplement 2: Eligibility criteria**

Eligibility for the EMIcompass RCT (Reininghaus et al., 2023) was assessed using observer-rated (Structured Clinical Interview for DSM-5 [SCID-5 (First et al., 2015)], Comprehensive Assessment of At-Risk Mental State [CAARMS (Yung et al., 2005)] and self-report (Kessler Psychological Distress Scale [K10 (Kessler et al., 2002; Kessler et al., 2005)] measures. The inclusion criteria were: (1) aged 14–25 years, (2) meeting criteria for stage 1a (K10 score≥20 (Kessler et al., 2002; Kessler et al., 2005)), stage 1b (CHARMS), or stage 2 (first episode of psychotic, bipolar, severe depressive, or severe anxiety disorder) based on the staging model by Hartmann et al. (2019), (3) high-stress reactivity assessed with a 2-item self-report measure (“Please think of the most unpleasant event in the last week: (a) How sad, disappointed, or angry have you been?, (b) Have you been sad, disappointed or angry because of your feelings?”) or the CAARMS subscale on impaired tolerance to everyday stress (Yung et al., 2005), (4) reduced positive affect (i.e., mean positive affect score <3.19 for men and <3.05 for women) or increased negative affect (i.e., mean negative affect score >1.81 for men and >1.75 for women (Breyer & Bluemke, 2016), based on normative scores (Breyer & Bluemke, 2016) of the Positive Affect and Negative Affect Scale [PANAS (Watson et al., 1988)], (5) willingness to participate, and (6) ability to provide written informed consent (or consent by parents or legal guardians in case of minors). Exclusion criteria were: (1) primary diagnosis of alcohol/substance abuse/dependence (SCID-5 (First et al., 2015)), 2) symptoms precipitated by an organic disease, (3) insufficient German to participate in trial processes, (4) diagnoses of a learning disability, and (5) acute suicidality (CAARMS score >4 (Yung et al., 2005)).

# **Supplement 3: Exploratory analyses of indirect effects of experimental condition on outcomes at post-intervention through candidate mechanisms**

To better understand the dynamic processes in possible mediating effects, we repeated our analyses as described in the Statistical analyses section with outcome variables measured at post-intervention (t_1_).

**The indirect effects of experimental condition on outcomes at post-intervention through putative mechanisms of change**

Findings on total, direct, and indirect effects of experimental condition and change in momentary resilience on psychological distress, clinical symptoms, and quality of life at post-intervention (T1) are displayed in Tables 9-13. We found no initial signals for indirect effects via pathways through the different mediators for psychological distress, clinical symptoms, and quality of life at post-intervention. We did observe total effects of change in emotional reactivity (β = 0.54, 95%CI = 0.04 – 1.02), change in momentary threat anticipation (β = 0.53, 95%CI = 0.03 – 1.08), change in resilience (β = 0.70, 95%CI = 0.13 – 1.32), and change in aberrant salience (β = -0.54, 95%CI = -0.90 – -0.29) on quality of life. We also found initial signals for direct effects of experimental condition on quality of life in the models with change in resilience (β = 0.67, 95%CI = 0.04 – 1.29) and change in aberrant salience (β = 0.48, 95%CI = 0.00 – 1.00). Moreover, we observed an effect of condition on change in aberrant salience in the models with all three outcomes (for psychological distress: β = -0.54, 95%CI = -0.90 – -0.28, for clinical symptoms: β = -0.56, 95%CI = -0.89 – -0.28, for quality of life: β = -0.52, 95%CI = -1.07 – -0.15). Last, we found associations between change in emotional reactivity (β = 0.44, 95%CI = 0.15 – 0.73) and change in momentary threat anticipation (β = 0.56, 95%CI = 0.22 – 0.84) on psychological distress.

| **Table S1.** *The indirect effects of experimental condition on psychological distress, clinical symptoms, and quality of life at post-intervention via pathways through change in stress reactivity^a,b^.* | | | | | | | | | | | |  |  |
| --- | --- | --- | --- | --- | --- | --- | --- | --- | --- | --- | --- | --- | --- |
|  | **Psychological distress** | | | **Clinical Symptoms** | | | **Quality of Life** | | | | | | |
|  | aβ^c^ | CI | P_M_^d^ | aβ | CI | P_M_ | aβ | | CI | | P_M_ | | |
| Total effect | 0.05 | -0.28 – 0.40 |  | -0.21 | -0.63 – 0.17 |  | 0.50 | -0.08 – 1.08 | |  | | | |
| Direct effect of condition on outcome | 0.05 | -0.30 – 0.42 |  | -0.21 | -0.62 – 0.19 |  | 0.52 | -0.02 – 1.11 | |  | | | |
| Effect of condition on mediator | -0.00 | -0.13 – 0.11 |  | 0.00 | -0.12 – 0.13 |  | 0.03 | -0.07 – 0.15 | |  | | | |
| Effect of mediator on outcome | 0.13 | -0.55 – 0.97 |  | -0.41 | -1.46 – 0.31 |  | -0.70 | -2.25 – 1.23 | |  | | | |
| Indirect effect | -0.00 | -0.05 – 0.04 | 0.01 | -0.00 | -0.08 – 0.06 | 0.00 | -0.02 | -0.23 – 0.06 | | 0.04 | | | |
| *Note*. ^a^ = n = 87 for psychological distress and clinical symptoms and 52 for quality of life. ^b^ =All analyses were adjusted for age, gender, and baseline levels of outcomes and mediator. ^c^ = standardized regression coefficient. ^d^ = Proportion mediated. ^e^ = exceeds 1.0 in case of suppression effects, i.e., if direct effect is opposite in sign to the indirect effect (Shrout & Bolger, 2002). | | | | | | | | | | | | |  |

| **Table S2.** *The indirect effects of experimental condition on psychological distress, clinical symptoms, and quality of life at post-intervention via pathways through change in emotional reactivity^a,b^.* | | | | | | | | | | |  | |  |
| --- | --- | --- | --- | --- | --- | --- | --- | --- | --- | --- | --- | --- | --- |
|  | **Psychological distress** | | | **Clinical Symptoms** | | | **Quality of Life** | | | | | | |
|  | aβ^c^ | CI | P_M_^d^ | aβ | CI | P_M_ | aβ | | CI | P_M_ | | | |
| Total effect | 0.04 | -0.34 – 0.43 |  | -0.27 | -0.67 – 0.19 |  | 0.54 | 0.04 – 1.02 | | | |  | |
| Direct effect of condition on outcome | 0.09 | -0.27 – 0.46 |  | -0.25 | -0.67 – 0.20 |  | 0.47 | -0.09 – 0.97 | | | |  | |
| Effect of condition on mediator | -0.12 | -0.38 – 0.15 |  | -0.13 | -0.39 – 0.17 |  | -0.18 | -0.53 – 0.13 | | | |  | |
| Effect of mediator on outcome | 0.44 | 0.15 – 0.73 |  | 0.13 | -0.20 – 0.38 |  | -0.37 | -0.76 – 0.02 | | | |  | |
| Indirect effect | -0.05 | -0.17 – 0.07 | 1.16^e^ | -0.02 | -0.14 – 0.02 | 0.06 | 0.07 | -0.03 – 0.29 | | | | 0.13 | |
| *Note*. ^a^ = n = 87 for psychological distress and clinical symptoms and 52 for quality of life. ^b^ =All analyses were adjusted for age, gender, and baseline levels of outcomes and mediator. ^c^ = standardized regression coefficient. ^d^ = Proportion mediated. ^e^ = exceeds 1.0 in case of suppression effects, i.e., if direct effect is opposite in sign to the indirect effect (Shrout & Bolger, 2002). | | | | | | | | | | | | |  |

| **Table S3.** *The indirect effects of experimental condition on psychological distress, clinical symptoms, and quality of life at post-intervention via pathways through change in threat anticipation^a,b^.* | | | | | | | | | | |  | |
| --- | --- | --- | --- | --- | --- | --- | --- | --- | --- | --- | --- | --- |
|  | **Psychological distress** | | | **Clinical Symptoms** | | | **Quality of Life** | | | | | |
|  | aβ^c^ | CI | P_M_^d^ | aβ | CI | P_M_ | aβ | | CI | P_M_ | | |
| **Momentary threat anticipation** | | |  |  |  |  |  |  | | | |  |
| Total effect | 0.07 | -0.31 – 0.45 |  | -0.20 | -0.65 – 0.30 |  | 0.53 | 0.03 – 1.08 | | | |  |
| Direct effect of condition on outcome | 0.18 | -0.18 – 0.53 |  | -0.36 | -0.63 – 0.41 |  | 0.40 | -0.15 – 1.06 | | | |  |
| Effect of condition on mediator | -0.20 | -0.46 – 0.04 |  | -0.22 | -0.45 – 0.02 |  | -0.32 | -0.67 – -0.02 | | | |  |
| Effect of mediator on outcome | 0.56 | 0.22 – 0.84 |  | 0.30 | -0.07 – 0.68 |  | -0.40 | -1.00 – 0.22 | | | |  |
| Indirect effect | -0.11 | -0.34 – 0.01 | 1.67^e^ | -0.06 | -0.27 – 0.01 | 0.32 | 0.13 | -0.03 – 0.49 | | | | 0.24 |
| **Threat anticipation measured with TAM** | | |  |  |  |  |  |  | | | |  |
| Total effect | 0.00 | -0.34 – 0.36 |  | -0.27 | -0.62 – 0.16 |  | 0.56 | 0.03 – 1.04 | | | |  |
| Direct effect of condition on outcome | -0.01 | -0.35 – 0.35 |  | -0.25 | -0.60 – 0.17 |  | 0.56 | 0.02 – 1.00 | | | |  |
| Effect of condition on mediator | -0.13 | -0.58 – 0.24 |  | -0.15 | -0.52 – 0.20 |  | 0.01 | -0.49 – 0.46 | | | |  |
| Effect of mediator on outcome | -0.05 | -0.24 – 0.16 |  | 0.14 | -0.11 – 0.39 |  | -0.31 | -0.75 – 0.06 | | | |  |
| Indirect effect | 0.01 | -0.02 – 0.10 | 18.41^e^ | -0.02 | -0.16 – 0.02 | 0.08 | -0.00 | -0.21 – 0.15 | | | | 0.01 |
| *Note*. ^a^ = n = 87 for the analyses with momentary threat anticipation with psychological distress and clinical symptoms and 52 for quality of life, n = 89 for the analyses with threat anticipation with psychological distress and clinical symptoms and 52 for quality of life. ^b^ = Adjusted for age, gender, and baseline levels of outcomes and mediator. ^c^ = standardized regression coefficient. ^d^ = Proportion mediated. *= exceeds 1.0 in case of suppression effects, i.e., if direct effect is opposite in sign to the indirect effect (Shrout & Bolger, 2002). **Table S4.** *The indirect effects of experimental condition on psychological distress, clinical symptoms, and quality of life at post-intervention via pathways through change in resilience^a,b^.* \| \| \| \| \| \| \| \| \| \| \| \| \|; \| **Psychological distress** \| \| \| **Clinical Symptoms** \| \| \| **Quality of Life** \| \| \| \| \| \|; \| aβ^c^ \| CI \| P_M_^d^ \| aβ \| CI \| P_M_ \| aβ \| \| CI \| \| P_M_ \| \|; Total effect \| -0.01 \| -0.46 – 0.44 \| \| -0.16 \| -0.67 – 0.37 \| \| 0.70 \| 0.13 – 1.32 \| \| \| \| \|; Direct effect of condition on outcome \| -0.02 \| -0.43 – 0.48 \| \| -0.08 \| -0.61 – 0.42 \| \| 0.67 \| 0.04 – 1.29 \| \| \| \| \|; Effect of condition on mediator \| 0.21 \| -0.20 – 0.62 \| \| 0.24 \| -0.17 – 0.69 \| \| 0.29 \| -0.14 – 0.74 \| \| \| \| \|; Effect of mediator on outcome \| -0.16 \| -0.48 – 0.16 \| \| -0.32 \| -0.66 – 0.01 \| \| 0.09 \| -0.33 – 0.47 \| \| \| \| \|; Indirect effect \| -0.03 \| -0.15 – 0.08 \| 3.07 \| -0.08 \| -0.39 – 0.03 \| 0.08 \| 0.03 \| -0.09 – 0.28 \| \| 0.04 \| \| \|; *Note*. ^a^ = n = 65 for psychological distress and clinical symptoms and 39 for quality of life. ^b^ =All analyses were adjusted for age, gender, and baseline levels of outcomes and mediator. ^c^ = standardized regression coefficient. ^d^ = Proportion mediated. ^e^ = exceeds 1.0 in case of suppression effects, i.e., if direct effect is opposite in sign to the indirect effect (Shrout & Bolger, 2002). \| \| \| \| \| \| \| \| \| \| \| \| \| | | | | | | | | | | | | |

| **Table S5.** *The indirect effects of experimental condition on psychological distress, clinical symptoms, and quality of life at post-intervention via pathways through change in aberrant salience^a,b^.* | | | | | | | | | | |  |  |  |  |
| --- | --- | --- | --- | --- | --- | --- | --- | --- | --- | --- | --- | --- | --- | --- |
|  | **Psychological distress** | | | **Clinical Symptoms** | | | | **Quality of Life** | | | | | | |
|  | aβ^c^ | CI | P_M_^d^ | aβ | CI | P_M_ | | aβ | | CI | | | P_M_ | |
| Total effect | 0.06 | -0.27 – 0.43 |  | -0.22 | -0.66 – 0.22 |  | | 0.56 | 0.09 – 1.06 | | | | |  |
| Direct effect of condition on outcome | 0.18 | -0.18 – 0.58 |  | -0.17 | -0.63 – 0.28 |  | | 0.48 | 0.00 – 1.00 | | | | |  |
| Effect of condition on mediator | -0.54 | -0.90 – -0.28 |  | -0.56 | -0.89 – -0.28 | |  | -0.52 | -1.07 – -0.15 | | | | |  |
| Effect of mediator on outcome | 0.21 | -0.06 – 0.57 |  | 0.09 | -0.24 – 0.32 |  | | -0.14 | -0.42 – 0.17 | | | | |  |
| Indirect effect | -0.12 | -0.33 – 0.01 | 1.97^e^ | -0.05 | -0.20 – 0.12 | 0.24 | | 0.07 | -0.05 – 0.28 | | | | | 0.13 |
| *Note*. ^a^ = n = 87 for psychological distress and clinical symptoms and 52 for quality of life. ^b^ =All analyses were adjusted for age, gender, and baseline levels of outcomes and mediator. ^c^ = standardized regression coefficient. ^d^ = Proportion mediated. ^e^ = exceeds 1.0 in case of suppression effects, i.e., if direct effect is opposite in sign to the indirect effect (Shrout & Bolger, 2002). | | | | | | | | | | | |  |  |  |

# **Supplement 4: Exploratory analyses with the different subscales of the WHOQOL-BREF**

To better understand possible mediating effects for domain-specific changes in quality of life, we repeated our analyses as described in the Statistical analyses section with the different subscales of the WHOQOL-BREF (i.e., physical health, psychological health, social relationships, and environment) measured at four-week follow-up (t_2_).

| **Table S6.** *The indirect effects of experimental condition on the subscales of quality of life at four-week follow-up via pathways through change in stress reactivity^a,b^.* | | | | | | | | | | | | | | |  |
| --- | --- | --- | --- | --- | --- | --- | --- | --- | --- | --- | --- | --- | --- | --- | --- |
|  | **Physical Health** | | | **Psychological Health** | | | **Social Relationships** | | | | | **Environment** | | | |
|  | aβ^c^ | CI | P_M_^d^ | aβ | CI | P_M_ | aβ | | CI | P_M_ | | aβ | CI | P_M_ | |
| Total effect | 0.41 | -0.08 – 0.85 |  | 0.59 | 0.06 – 1.05 |  | 0.43 | -0.03 – 0.96 | | |  | -0.49 | -1.07 – 0.02 |  | |
| Direct effect of condition on outcome | 0.40 | -0.10 – 0.87 |  | 0.59 | 0.04 – 1.06 |  | 0.42 | -0.03 – 0.97 | | |  | -0.46 | -1.07 – 0.01 |  | |
| Effect of condition on mediator | 0.04 | -0.06 – 0.15 |  | 0.04 | -0.06 – 0.16 |  | 0.03 | -0.07 – 0.14 | | |  | 0.04 | -0.08 – 0.15 |  | |
| Effect of mediator on outcome | 0.11 | -1.02 – 1.24 |  | -0.17 | -1.37 – 0.79 |  | 0.43 | -0.88 – 1.36 | | |  | -0.81 | -2.64 – 0.66 |  | |
| Indirect effect | 0.00 | -0.05 – 0.16 | 0.01 | -0.01 | -0.16 – 0.04 | -0.01 | 0.01 | -0.03 – 0.21 | | | 0.03 | -0.03 | -0.42 – 0.04 | 0.06 | |
| *Note*. ^a^ = n = 87 for psychological distress and clinical symptoms and 52 for quality of life. ^b^ =All analyses were adjusted for age, gender, and baseline levels of outcomes and mediator. ^c^ = standardized regression coefficient. ^d^ = Proportion mediated. ^e^ = exceeds 1.0 in case of suppression effects, i.e., if direct effect is opposite in sign to the indirect effect (Shrout & Bolger, 2002). | | | | | | | | | | | | | | |  |

| **Table S7.** *The indirect effects of experimental condition on the subscales of quality of life at four-week follow-up via pathways through change in emotional reactivity^a,b^.* | | | | | | | | | | | | | | |
| --- | --- | --- | --- | --- | --- | --- | --- | --- | --- | --- | --- | --- | --- | --- |
|  | **Physical Health** | | | **Psychological Health** | | | **Social Relationships** | | | | | **Environment** | | |
|  | aβ^c^ | CI | P_M_^d^ | aβ | CI | P_M_ | aβ | | CI | P_M_ | | aβ | CI | P_M_ |
| Total effect | 0.45 | -0.00 – 0.94 |  | 0.61 | 0.08 – 1.10 |  | 0.42 | -0.08 – 0.97 | | |  | -0.44 | -1.04 – 0.05 |  |
| Direct effect of condition on outcome | 0.45 | -0.00 – 0.94 |  | 0.57 | 0.07 – 1.11 |  | 0.42 | -0.09 – 0.98 | | |  | -0.50 | -1.08 – 0.02 |  |
| Effect of condition on mediator | -0.17 | -0.51 – 0.12 |  | -0.18 | -0.50 – 0.10 |  | -0.16 | -0.51 – 0.17 | | |  | -0.15 | -0.47 – 0.16 |  |
| Effect of mediator on outcome | -0.00 | -0.62 – 0.44 |  | -0.23 | -0.74 – 0.24 |  | 0.01 | -0.45 – 0.54 | | |  | -0.38 | -1.04 – 0.10 |  |
| Indirect effect | 0.00 | -0.15 – 0.13 | 0.00 | 0.04 | -0.02 – 0.27 | 0.07 | -0.00 | -0.14 – 0.09 | | | -0.00 | 0.06 | -0.03 – 0.33 | -0.01 |
| *Note*. ^a^ = n = 52. ^b^ =All analyses were adjusted for age, gender, and baseline levels of outcomes and mediator. ^c^ = standardized regression coefficient. ^d^ = Proportion mediated. ^e^ = exceeds 1.0 in case of suppression effects, i.e., if direct effect is opposite in sign to the indirect effect (Shrout & Bolger, 2002). | | | | | | | | | | | | | | |

| **Table S8.** *The indirect effects of experimental condition on the subscales of quality of life at four-week follow-up via pathways through change in threat anticipation^,b^.* | | | | | | | | | | | | | |  |
| --- | --- | --- | --- | --- | --- | --- | --- | --- | --- | --- | --- | --- | --- | --- |
|  | **Physical Health** | | | **Psychological Health** | | | **Social Relationships** | | | | | **Environment** | | |
|  | aβ^c^ | CI | P_M_^d^ | aβ | CI | P_M_ | aβ | CI | | P_M_ | | aβ | CI | P_M_ |
| **Threat anticipation measured with EMA** | | | | | | | | | | | | | |  |
| Total effect | 0.45 | -0.07 – 0.95 |  | 0.62 | 0.10 – 1.13 |  | 0.43 | | -0.07 – 0.93 | |  | -0.45 | -1.07 – 0.05 |  |
| Direct effect of condition on outcome | 0.38 | -0.12 – 0.87 |  | 0.56 | 0.04 – 1.16 |  | 0.46 | | -0.09 – 1.06 | |  | -0.56 | -1.19 – 0.01 |  |
| Effect of condition on mediator | -0.31 | -0.64 – 0.00 |  | -0.33 | -0.71 – 0.02 |  | -0.27 | | -0.63 – 0.02 | |  | -0.29 | -0.59 – 0.06 |  |
| Effect of mediator on outcome | -0.25 | -0.84 – 0.31 |  | -0.16 | -0.61 – 0.28 |  | 0.10 | | -0.30 – 0.62 | |  | -0.36 | -0.93 – 0.20 |  |
| Indirect effect | 0.08 | -0.05 – 0.43 | 0.02 | 0.05 | -0.05 – 0.31 | 0.08 | -0.03 | | -0.28 – 0.06 | | -0.07 | 0.10 | -0.03 – 0.39 | -0.23 |
| **Threat anticipation measured with TAM** | | | | | | | | | | | | | |  |
| Total effect | 0.47 | -0.02 – 1.00 |  | 0.61 | 0.13 – 1.05 |  | 0.41 | | -0.05 – 0.89 | |  | -0.43 | -1.02 – 0.14 |  |
| Direct effect of condition on outcome | 0.48 | -0.02 – 0.99 |  | 0.61 | 0.12 – 1.05 |  | 0.41 | | -0.04 – 0.90 | |  | -0.43 | -1.00 – 0.11 |  |
| Effect of condition on mediator | 0.02 | -0.45 – 0.50 |  | 0.00 | -0.44 – 0.48 |  | 0.00 | | -0.50 – 0.42 | |  | 0.01 | -0.46 – 0.50 |  |
| Effect of mediator on outcome | -0.18 | -0.60 – 0.07 |  | -0.05 | -0.36 – 0.19 |  | -0.20 | | -0.39 – 0.17 | |  | -0.31 | -0.81 – 0.12 |  |
| Indirect effect | -0.00 | -0.16 – 0.08 | -0.01 | -0.00 | -0.06 – 0.08 | -0.00 | -0.00 | | -0.11 – 0.10 | | -0.00 | -0.00 | -0.23 – 0.15 | 0.00 |
| *Note*. ^a^ = n = 52. ^b^ =All analyses were adjusted for age, gender, and baseline levels of outcomes and mediator. ^c^ = standardized regression coefficient. ^d^ = Proportion mediated. ^e^ = exceeds 1.0 in case of suppression effects, i.e., if direct effect is opposite in sign to the indirect effect (Shrout & Bolger, 2002). | | | | | | | | | | | | | |  |

| **Table S9.** *The indirect effects of experimental condition on the subscales of quality of life at four-week follow-up via pathways through change in resilience^a,b^.* | | | | | | | | | | | | | | |
| --- | --- | --- | --- | --- | --- | --- | --- | --- | --- | --- | --- | --- | --- | --- |
|  | **Physical Health** | | | **Psychological Health** | | | **Social Relationships** | | | | | **Environment** | | |
|  | aβ^c^ | CI | P_M_^d^ | aβ | CI | P_M_ | aβ | | CI | P_M_ | | aβ | CI | P_M_ |
| Total effect | 0.68 | 0.14 – 1.25 |  | 0.96 | 0.40 – 1.38 |  | 0.56 | 0.10 – 1.11 | | |  | -0.23 | -0.80 – 0.38 |  |
| Direct effect of condition on outcome | 0.67 | 0.14 – 1.24 |  | 0.86 | 0.30 – 1.34 |  | 0.43 | 0.01 – 0.88 | | |  | -0.21 | -0.79 – 0.43 |  |
| Effect of condition on mediator | 0.29 | -0.16 – 0.75 |  | 0.35 | -0.11 – 0.85 |  | 0.34 | -0.10 – 0.89 | | |  | 0.32 | -0.11 – 0.76 |  |
| Effect of mediator on outcome | 0.07 | -0.28 – 0.52 |  | 0.30 | 0.05 – 0.59 |  | 0.39 | 0.05 – 0.92 | | |  | -0.04 | -0.56 – 0.46 |  |
| Indirect effect | 0.02 | -0.07 – 0.24 | 0.03 | 0.10 | -0.01 – 0.38 | 0.11 | 0.13 | -0.02 – 0.55 | | | 0.24 | -0.01 | -0.28 – 0.14 | 0.06 |
| *Note*. ^a^ = n = 52. ^b^ =All analyses were adjusted for age, gender, and baseline levels of outcomes and mediator. ^c^ = standardized regression coefficient. ^d^ = Proportion mediated. ^e^ = exceeds 1.0 in case of suppression effects, i.e., if direct effect is opposite in sign to the indirect effect (Shrout & Bolger, 2002). | | | | | | | | | | | | | | |

| **Table S10.** *The indirect effects of experimental condition on the subscales of quality of life at four-week follow-up via pathways through change in aberrant salience^,b^.* | | | | | | | | | | | | | | |
| --- | --- | --- | --- | --- | --- | --- | --- | --- | --- | --- | --- | --- | --- | --- |
|  | **Physical Health** | | | **Psychological Health** | | | **Social Relationships** | | | | | **Environment** | | |
|  | aβ^c^ | CI | P_M_^d^ | aβ | CI | P_M_ | aβ | CI | | P_M_ | | aβ | CI | P_M_ |
| Total effect | 0.47 | -0.04 – 1.04 |  | 0.61 | 0.09 – 1.10 |  | 0.41 | | -0.04 – 0.92 | |  | -0.43 | -0.98 – 0.14 |  |
| Direct effect of condition on outcome | 0.49 | -0.09 – 1.08 |  | 0.51 | -0.09 – 1.07 |  | 0.39 | | -0.11 – 0.97 | |  | -0.40 | -1.05 – 0.21 |  |
| Effect of condition on mediator | -0.50 | -1.04 – -0.14 |  | -0.53 | -1.06 – -0.14 |  | -0.51 | | -0.98 – -0.13 | |  | -0.49 | -0.99 – -0.13 |  |
| Effect of mediator on outcome | 0.04 | -0.39 – 0.34 |  | -0.19 | -0.51 – 0.24 |  | -0.04 | | -0.37 – 0.25 | |  | 0.06 | -0.31 – 0.49 |  |
| Indirect effect | -0.02 | -0.22 – 0.15 | -0.04 | 0.10 | -0.06 – 0.36 | 0.16 | 0.02 | | -0.15 – 0.19 | | 0.05 | -0.03 | -0.29 – 0.12 | 0.06 |
| *Note*. ^a^ = n = 52. ^b^ =All analyses were adjusted for age, gender, and baseline levels of outcomes and mediator. ^c^ = standardized regression coefficient. ^d^ = Proportion mediated. ^e^ = exceeds 1.0 in case of suppression effects, i.e., if direct effect is opposite in sign to the indirect effect (Shrout & Bolger, 2002). | | | | | | | | | | | | | | |

# **Supplement 5: Sensitivity analyses with different conceptualisations of change in stress reactivity**

To reflect consistency of our analyses with results of the EMIcompass RCT (Reininghaus et al., 2023), we performed sensitivity analyses using different conceptualisations of change in stress reactivity and regression analyses to examine the effect of condition on stress reactivity.

1) Using change in stress reactivity from baseline to four-week follow-up (δ4-week follow-up–baseline) as mediator

| **Table S11.** *The indirect effects of experimental condition on psychological distress, clinical symptoms, and quality of life at four-week follow-up via pathways through change in stress reactivity (δ4-week follow-up–baseline)^a,b^.* | | | | | | | | | | | |  |  |
| --- | --- | --- | --- | --- | --- | --- | --- | --- | --- | --- | --- | --- | --- |
|  | **Psychological distress** | | | **Clinical Symptoms** | | | **Quality of Life** | | | | | | |
|  | aβ^c^ | CI | P_M_^d^ | aβ | CI | P_M_ | aβ | | CI | P_M_ | | | |
| Total effect | -0.02 | -0.40 – 0.36 |  | 0.26 | -0.14 – 0.69 |  | 0.28 | -0.36 – 0.81 | | |  | | |
| Direct effect of condition on outcome | 0.00 | -0.37 – 0.37 |  | 0.24 | -0.12 – 0.68 |  | 0.30 | -0.32 – 0.84 | | |  | | |
| Effect of condition on mediator | -0.05 | -0.19 – 0.07 |  | -0.04 | -0.17 – 0.09 |  | 0.02 | -0.08 – 0.14 | | |  | | |
| Effect of mediator on outcome | 0.50 | -0.33 – 1.31 |  | -0.51 | -1.47 – 1.00 |  | -0.66 | -2.25 – 0.62 | | |  | | |
| Indirect effect | -0.02 | -0.21 – 0.02 | 1.14* | 0.02 | -0.04 – 0.18 | 0.07 | -0.02 | -0.25 – 0.04 | | | 0.05 | | |
| *Note*. ^a^ = n = 87 for psychological distress and clinical symptoms and 52 for quality of life. ^b^ =All analyses were adjusted for age, gender, and baseline levels of outcomes and mediator. ^c^ = standardized regression coefficient. ^d^ = Proportion mediated. ^e^ = exceeds 1.0 in case of suppression effects, i.e., if direct effect is opposite in sign to the indirect effect (Shrout & Bolger, 2002). | | | | | | | | | | | | |  |

2) Using change in stress reactivity from post-intervention to four-week follow-up (δ4-week follow-up–post-intervention) as mediator

| **Table S12.** *The indirect effects of experimental condition on psychological distress, clinical symptoms, and quality of life at four-week follow-up via pathways through change in stress reactivity (δ4-week follow-up–post-intervention)^a,b^.* | | | | | | | | | | |  | |  |
| --- | --- | --- | --- | --- | --- | --- | --- | --- | --- | --- | --- | --- | --- |
|  | **Psychological distress** | | | **Clinical Symptoms** | | | **Quality of Life** | | | | | | |
|  | aβ^c^ | CI | P_M_^d^ | aβ | CI | P_M_ | aβ | | CI | P_M_ | | | |
| Total effect | -0.02 | -0.40 – 0.40 |  | 0.25 | -0.15 – 0.70 |  | 0.28 | -0.33 – 0.82 | | | |  | |
| Direct effect of condition on outcome | -0.05 | -0.46 – 0.35 |  | 0.22 | -0.17 – 0.65 |  | 0.28 | -0.35 – 0.83 | | | |  | |
| Effect of condition on mediator | -0.06 | -0.15 – 003 |  | -0.05 | -0.14 – 0.03 |  | -0.01 | -0.13 – 0.13 | | | |  | |
| Effect of mediator on outcome | -0.57 | -1.48 – 0.64 |  | -0.62 | -1.80 – 0.42 |  | -0.35 | -1.64 – 1.07 | | | |  | |
| Indirect effect | 0.03 | -0.02 – 0.19 | 2.17* | 0.03 | -0.01 – 0.22 | 0.13 | 0.01 | -0.07 – 0.18 | | | | 0.01 | |
| *Note*. ^a^ = n = 87 for psychological distress and clinical symptoms and 52 for quality of life. ^b^ =All analyses were adjusted for age, gender, and baseline levels of outcomes and mediator. ^c^ = standardized regression coefficient. ^d^ = Proportion mediated. ^e^ = exceeds 1.0 in case of suppression effects, i.e., if direct effect is opposite in sign to the indirect effect (Shrout & Bolger, 2002). | | | | | | | | | | | | | |

3) Regression analyses to examine effects of condition on level stress reactivity (i.e., using aggregated EMA data) measured at post-intervention and follow-up.

| **Table S13.** *Effects of condition on stress reactivity measured at post-intervention and follow-up.* | | |
| --- | --- | --- |
|  | β | *CI* |
| **Stress reactivity measured at post-intervention** | | |
| Condition | 0.02 | -0.11 – 0.14 |
| Baseline stress reactivity | 0.97 | 0.89 – 1.04 |
| **Stress reactivity measured at follow-up** | | |
| Condition | -0.04 | -0.16 – 0.07 |
| Baseline stress reactivity | 0.96 | 0.87 – 1.02 |
| *Note*. Stress reactivity measured at post-intervention and measured at follow up were each entered as dependent variable and condition and stress reactivity measured at baseline were entered as independent variables. All measures were z-standardized. CI = 95% confidence interval. N = 85 observations. | | |

| **Table S14.** *Intercorrelation matrix for all candidate mechanisms of change^a^ and outcome variables^b^ in our main analyses.* | | | | | | | | | | |
| --- | --- | --- | --- | --- | --- | --- | --- | --- | --- | --- |
|  | Change in stress reactivity | Change in emotional reactivity | Change in threat anticipation measured with EMA | Change in threat anticipation measured with TAM | Change in resilience | Change in aberrant salience | Psychological distress | Clinical symptoms | Quality of life | Prodromal symptoms |
| Change in stress reactivity | 1.0 |  |  |  |  |  |  |  |  |  |
| Change in emotional reactivity | 0.59** | 1.0 |  |  |  |  |  |  |  |  |
| Change in threat anticipation measured with EMA | 0.41** | 0.63** | 1.0 |  |  |  |  |  |  |  |
| Change in threat anticipation measured with TAM | -0.02 | 0.13 | -0.05 | 1.0 |  |  |  |  |  |  |
| Change in resilience | 0.09 | -0.12 | -0.26* | 0.10 | 1.0 |  |  |  |  |  |
| Change in aberrant salience | 0.09 | 0.52** | 0.28* | 0.34* | -0.07 | 1.0 |  |  |  |  |
| Psychological distress | 0.17 | 0.34* | 0.30* | 0.01 | -0.04 | 0.10 | 1.0 |  |  |  |
| Clinical symptoms | -0.08 | 0.18 | 0.23* | 0.06 | -0.02 | 0.07 | 0.33* | 1.0 |  |  |
| Quality of life | -0.00 | -0.15 | -0.23 | -0.10 | 0.21 | -0.07 | -0.57** | -0.22 | 1.0 |  |
| Prodromal symptoms | -0.10 | 0.11 | 0.09 | 0.10 | -0.14 | -0.06 | 0.35** | 0.41** | -0.31* | 1.0 |
| *Note*. * = p < 0.05, ** = p < 0.001. ^a^ = For each candidate mechanism of change, a difference score was calculated (δpost-intervention–baseline) to index change in the respective mechanism. ^b^ = The outcome variables (i.e., psychological distress, clinical symptoms, quality of life, and prodromal symptoms) were assessed at four-week follow up. | | | | | | | | | | |

# **Supplement 6: Intercorrelations and multicollinearity diagnostics**

We evaluated multicollinearity by calculating variance inflation factors from the corresponding linear regression models. All variance inflation factors ranged from 1.04 to 1.53, which is below commonly used thresholds, indicating no evidence of problematic multicollinearity (Dormann et al., 2013; Hair et al., 2019; Shrestha, 2020).

# **References**

Breyer, B., & Bluemke, M. (2016). *Deutsche version der positive and negative affect schedule PANAS (GESIS panel)* <https://nbn-resolving.org/urn:nbn:de:0168-ssoar-65680-6>

Dormann, C. F., Elith, J., Bacher, S., Buchmann, C., Carl, G., Carré, G., Marquéz, J. R. G., Gruber, B., Lafourcade, B., & Leitão, P. J. (2013). Collinearity: a review of methods to deal with it and a simulation study evaluating their performance. *Ecography*, *36*(1), 27-46.

First, M., Williams, J., Karg, R., & Spitzer, R. (2015). Structured Clinical Interview for DSM-5—Research Version (SCID-5 for DSM5, Research Version; SCID-5 for DSM-5, Research Version; SCID-5-RV). *VA: American Psychiatric Association*, 1-94.

Hair, J. F., Risher, J. J., Sarstedt, M., & Ringle, C. M. (2019). When to use and how to report the results of PLS-SEM. *European business review*, *31*(1), 2-24.

Hartmann, J. A., Nelson, B., Spooner, R., Amminger, G. P., Chanen, A., Davey, C. G., McHugh, M., Ratheesh, A., Treen, D., Yuen, H. P., & McGorry, P. D. (2019). Broad clinical high-risk mental state (CHARMS): Methodology of a cohort study validating criteria for pluripotent risk. *Early Intervention in Psychiatry*, *13*(3), 379-386. <https://doi.org/https://doi.org/10.1111/eip.12483>

Kessler, R. C., Andrews, G., Colpe, L. J., Hiripi, E., Mroczek, D. K., Normand, S.-L., Walters, E. E., & Zaslavsky, A. M. (2002). Short screening scales to monitor population prevalences and trends in non-specific psychological distress. *Psychological medicine*, *32*(6), 959-976.

Kessler, R. C., Berglund, P., Demler, O., Jin, R., Merikangas, K. R., & Walters, E. E. (2005). Lifetime prevalence and age-of-onset distributions of DSM-IV disorders in the National Comorbidity Survey Replication. *Archives of general psychiatry*, *62*(6), 593-602.

Reininghaus, U., Paetzold, I., Rauschenberg, C., Hirjak, D., Banaschewski, T., Meyer-Lindenberg, A., Boecking, B., Boehnke, J. R., & Schick, A. (2023). Effects of a Novel, Transdiagnostic, Hybrid Ecological Momentary Intervention for Improving Resilience in Youth (EMIcompass): findings from an exploratory randomized controlled trial. *Schizophrenia Bulletin*, *49*(3), 592-604. <https://doi.org/https://doi.org/10.1093/schbul/sbac212>

Shrestha, N. (2020). Detecting multicollinearity in regression analysis. *American journal of applied mathematics and statistics*, *8*(2), 39-42.

Shrout, P. E., & Bolger, N. (2002). Mediation in experimental and nonexperimental studies: new procedures and recommendations. *Psychological Methods*, *7*(4), 422.

Watson, D., Clark, L. A., & Tellegen, A. (1988). Development and validation of brief measures of positive and negative affect: the PANAS scales. *Journal of personality and social psychology*, *54*(6), 1063-1070.

Yung, A., Yuen, H., McGorry, P., Phillips, L., Kelly, D., & Dell'Olio, M. (2005). Mapping the onset of psychosis-the Comprehensive Assessment of At Risk Mental States (CAARMS). *Australia and New Zealand Journal of Psychiatry*, *24*, 964-971.
